# Simplified Footprint-Free Cas9/CRISPR Editing of Cardiac-Associated Genes in Human Pluripotent Stem Cells

**DOI:** 10.1089/scd.2017.0268

**Published:** 2018-03-15

**Authors:** Alexander Kondrashov, Minh Duc Hoang, James G.W. Smith, Jamie R. Bhagwan, Gary Duncan, Diogo Mosqueira, Maria Barbadillo Munoz, Nguyen T.N. Vo, Chris Denning

**Affiliations:** Department of Stem Cell Biology, Centre of Biomolecular Sciences, University of Nottingham, Nottingham, United Kingdom.

**Keywords:** Cas9/CRISPR, PiggyBac, gene editing, human pluripotent stem cells, genetic disease modeling, cardiomyocytes

## Abstract

Modeling disease with human pluripotent stem cells (hPSCs) is hindered because the impact on cell phenotype from genetic variability between individuals can be greater than from the pathogenic mutation. While “footprint-free” Cas9/CRISPR editing solves this issue, existing approaches are inefficient or lengthy. In this study, a simplified *PiggyBac* strategy shortened hPSC editing by 2 weeks and required one round of clonal expansion and genotyping rather than two, with similar efficiencies to the longer conventional process. Success was shown across four cardiac-associated loci (*ADRB2*, *GRK5*, *RYR2*, and *ACTC1*) by genomic cleavage and editing efficiencies of 8%–93% and 8%–67%, respectively, including mono- and/or biallelic events. Pluripotency was retained, as was differentiation into high-purity cardiomyocytes (CMs; 88%–99%). Using the GRK5 isogenic lines as an exemplar, chronic stimulation with the β-adrenoceptor agonist, isoprenaline, reduced beat rate in hPSC-CMs expressing GRK5-Q41 but not GRK5-L41; this was reversed by the β-blocker, propranolol. This shortened, footprint-free approach will be useful for mechanistic studies.

## Introduction

Human pluripotent stem cells (hPSCs) comprise both human embryonic stem cells (hESCs), derived from the inner cell mass of the preimplantation embryo, and human-induced pluripotent stem cells (hiPSCs), derived by epigenetic reprogramming of somatic cells [1]. It is now well established that hPSCs are an important modality for biomedicine, with application ranging from understanding human development through to use of their differentiated progeny in safety assessment of drugs, accelerating drug use toward clinic and modeling genetic disease [1]. Suitability in several clinical trials has been, or is being evaluated, including for spinal cord injury, macular degeneration, and heart disease [2]. A difficulty that has emerged for the in vitro assays is that genetic variation between unrelated individuals may cause greater phenotypic differences than do the disease-associated polymorphism(s) [3]. Therefore, creation of isogenic pairs, wherein only the polymorphism of interest differs between lines, is now considered the gold standard. While the number of reports using conventional gene targeting in hPSC is low, the advent of nuclease-mediated targeting, particularly with Cas9/CRISPR, has made precise modification of the genome relatively routine [1].

Despite these advances, difficulties still remain in gene editing of hPSCs. Making single base-pair substitutions is technologically challenging when compared to, for example, gene knockouts, where libraries of guide RNAs (gRNAs) are being used in functional genome-wide screens [4]. An important consideration for editing is that, other than the desired polymorphic changes, the level of genome modification postgene-edited hPSC line should be minimal. This is because residual footprints left behind after targeting can alter or abolish neighboring gene expression [1,5,6]. This advocates the use of footprint-free or scarless approaches.

One route to achieving footprint-free editing is via the delivery of ribonucleoprotein combinations that comprise recombinant Cas9 protein, in vitro transcribed gRNA and a ∼50–150 base single-stranded DNA oligonucleotide (ssODN) template, which carries the polymorphic change(s) of interest [7]. We demonstrated the utility of this approach by modifying the *ADRB2* locus, which encodes the β2-adrenocetor [1], while others have altered additional loci [7,8]. Although this route is attractive and less toxic than plasmid approach [7], it requires high transfection rates of large complexes, which can be difficult in sensitive cells such as hPSCs. The lack of a drug selection marker also means that considerable screening effort is needed to identify positive clones. An alternative to achieving seamless editing by using ssODNs as a template is via a system termed “*CORRECT*” [9]; however, this requires two sequential clonal selection/expansion steps.

**FIG. 1.**
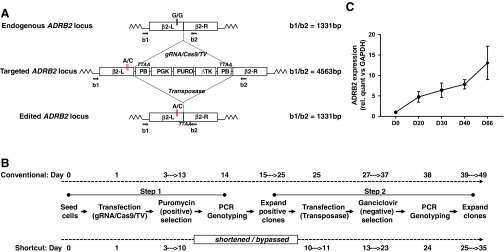
*PiggyBac* targeting at the *ADRB2* locus. **(A)** Shows a schematic of the *ADRB2* locus structure before targeting, after insertion of the *PiggyBac* positive–negative selection cassette and after cassette excision. The *black* (G/G) and *red* (A/C) *vertical lines* indicate the location of the polymorphic changes induced at bases 46 and 79. Primer locations (b1, b2) for genotyping are indicated, along with PCR product sizes. β2-L and β2-R indicate the left and right regions of homology, each of 1 kb in length. **(B)** Shows the time line of the conventional two-step *PiggyBac* targeting approach (*upper*) and the simplified approach (*lower*). In **(C)**, expression of the *ADRB2* gene was evaluated by quantitative real-time PCR in undifferentiated hPSCs (U) and through a 66-day timecourse of directed monolayer differentiation to CMs; beating sheets appeared from between d8-12. Data are mean ± SEM; *n* = 4. CM, cardiomyocyte; hPSCs, human pluripotent stem cells; PB, *PiggyBac*; PCR, polymerase chain reaction; PGK, phosphoglycerate kinase promoter; PURO, puromycin-N-acetyltransferase; TK, thymidine kinase; TV, targeting vector. Color images available online at www.liebertpub.com/scd

An alternative for footprint-free editing is the *PiggyBac* transposon system [10], although this does require a *TTAA* quadranucleotide site for recombination (Fig. 1). In this approach, a targeting vector contains a positive–negative drug selection cassette (eg, Puro-ΔTK; Fig. 1A) that is flanked by *PiggyBac* recombination sites. In turn, these components are flanked by regions of up to 1 kb in length that are homologous to the endogenous target locus, thus enabling recombination between template and genome. The desired polymorphism(s) is carried within one arm of homology.

Experimentally, the approach is implemented via two sequential steps. First, the targeting vector is cotransfected with plasmids carrying guide RNA and Cas9 to promote genomic cleavage and insertion via homology-directed repair into the locus of interest. Survival during positive selection with antibiotics (eg, puromycin) identifies the hPSC clones that express the cassette, which are then picked, expanded, and genotyped (Fig. 1B). Second, antibiotic-resistant hPSCs are transfected with a plasmid expressing transposase, which induces internal recombination between *PiggyBac* sites, excision of the selection cassette, and reconstitution of a footprint-free locus (Fig. 1B). Colonies that fail to excise the cassette continue to express ΔTK and hence are negatively selected against by the prodrugs, ganciclovir or fialuridin. This leaves the surviving colonies, which can be picked, expanded, and genotyped for a second time.

Several reports have described the successful use of this *PiggyBac* approach in hPSC [11–13]. Nevertheless, the requirement for two rounds of clonal selection and genotyping over a lengthy timeline is problematic. Particularly for hPSCs, the number of cumulative population doublings correlates genetic [14] and epigenetic [15,16] instability, thereby affecting their downstream applications [17]. Similarly, in mouse iPSCs, genetic instability has been reported within as few as 4–6 passages [18]. Thus, processes that enable gene editing in shorter timelines would be beneficial [19].

In this report, we adapted a footprint-free *PiggyBac*-based Cas9/CRISPR gene editing strategy to both simplify and shorten the process. Only one round of clonal selection and genotyping is needed, reducing the process from 49 to 35 days, a 25%–30% time saving that equates to ∼14 population doublings in hPSCs. We have demonstrated the utility of this simplified approach by making single or dual polymorphic changes to four cardiac-related genes, *ADRB2*, *GRK5*, *RYR2*, and *ACTC1.* For each of the engineered hPSC lines created, we showed that the cells retained expression of pluripotency markers, a stable karyotype, and the ability to differentiate at high efficiency into beating cardiomyocytes (CM) that express α-actinin. As an exemplar, we showed significant differences in functional consequence between isogenic pairs of hiPSC-CMs that carry GRK5-L41 or GRK5-Q41 polymorphisms in response to chronic β-adrenergic stimulation and β-blocker rescue. Thus, the approach described provides a simplified and abbreviated route toward mechanistic understanding of how single polymorphic variants alter heart function.

## Materials and Methods

### Cell culture

All cultures were at 37°C at 5% CO_2_ in a humidified atmosphere. Unless otherwise stated, all reagents were from ThermoFisher. HUES7 hESCs were gifted by Chad Cowan and Doug Melton at the Harvard Stem Cell Institute. Fibroblasts were derived under ethical consent from individual with the genotypes *RYR2^6739C/T^* (NRES Committee East Midlands–Nottingham 2 approval 09/H0408/74) and *ACTC1^301G/G^* (Biomedical Institute of A Coruna, INIBIC). Reprogramming to hiPSCs was via CytoTune 2.0 (ThermoFisher), according to the manufacturer's instructions. Culture was in E8 medium on Matrigel, although processes could also be completed in hESC medium conditioned using mouse embryonic fibroblasts [20]. In the first 4–5 passages after reprogramming, cell harvesting was done using 0.5 mM EDTA and thereafter with accutase.

### Transfection optimization

For transfection and electroporation experiments, hPSCs were seeded at 3 × 10^5^ cells/well of the Matrigel-coated 6-well plate or resuspended cells at 2 × 10^5^ cell/well/transfection condition in Nucleocuvette Strip (16 wells), respectively. Plasmids were transfected into hPSCs using FuGene HD transfection reagent (E2311; Promega) following the manufacturer's instructions, using a ratio between reagent and plasmid DNA of 4:1. To optimize the electroporation using the Amaxa 4D system (Lonza), pmaxGFP plasmid provided in the Lonza Amaxa 4D Kit was transfected into hPSCs with human stem cell P3 solution (programs: CA-137, CB-150, CD-1118, CE-118, CM-113, DC-100, DN-100, as recommended by the manufacturer's protocol). The green fluorescent protein signal was captured using Operetta High-Content Imaging System (Perkin Elmer) and analyzed using Harmony High-Content Imaging Software.

### Targeting vector construction

The *ADRB2* targeting vector was constructed via Gibson assembly by using Gibson Assembly Master Mix (E2611S NEB). Overlapping fragments were produced by polymerase chain reaction (PCR) (GoTaq polymerase; Promega) for three inserts: Dual drug selection cassette (Puro-ΔTK) flanked by *PiggyBac* recombination sites and the left and right homology regions for *ADRB2* (∼1 kb upstream and ∼1 kb downstream of the locus cut site). Primers used are shown in Supplementary Table S1 (Supplementary Data are available online at www.liebertpub.com/scd). An EcoRV-digested pBluescript backbone plasmid sequence was used as the fourth DNA fragment in the Gibson assembly. A 20 μL reaction containing 0.24 pmol of each insert, 0.08 pM of Bluescript backbone, and 1 × Gibson Assembly^®^ Master Mix (NEB) was heated at 50°C for 60 min. Subsequent transformation into Top10 competent cells and colony sequencing identified correctly assembled plasmids. The same approach was used to generate the *GRK5*, *ACTC1*, and *RYR2* targeting constructs.

### Gene targeting in hPSCs

FuGene HD (Promega) transfection required seeding of 3 × 10^5^ hPSCs into each well of a Matrigel-coated 6-well plate. Twenty-four hours later, cells were transfected 3.3 μg of CRISPR plasmid components (targeting plasmid, gRNA, Cas9). For Amaxa 4D nucleofection (Lonza), 3 × 10^6^ hPSCs and 3 μg of CRISPR plasmid components were used with P3 solution, program CA-137. Transfected and nucleofected cells were maintained in E8 medium on Matrigel (hESC medium conditioned using mouse embryonic fibroblasts [20] could also be used). Twenty-four hours posttransfection, medium was supplemented with puromycin (0.25–7.5 μg/mL; cell line dependent) for positive selection of clones up to 2 weeks. The puromycin-positive clones were then harvested and expanded as described in the cell culture section. For cassette excision, cells were seeded at 3 × 10^5^ cells/well of a Matrigel-coated 6-well plate before delivering transposase plasmid by transfection (3 μg) using FuGene HD transfection as described above. Cells were reseeded to 10 cm dishes, incubated for 2–3 days to allow recombination by transposase and then exposed to medium containing ganciclovir (2 μg/mL) for negative selection of *PiggyBac* excision. Approximately 7–10 days later, clones were manually dissected and genotyped using primers shown in Supplementary Table S1. See this Table and also Fig. 3 for location of primers to test for off-target and random integration events. Real-time quantitative PCR (qPCR) to the ampicillin gene was conducted by GoTag^®^ qPCR Master Mix (No. A6001; Promega) on Applied Biosystems SDS 7500 Fast Real-time PCR template for 45 cycles. Melting curves were obtained for all experimental runs. Relative expression of genes was calculated and expressed as 2−ΔΔCt, normalized using *18S*.

### Characterization of hPSC

#### CM differentiation

Undifferentiated hPSCs were seeded onto Matrigel-coated dishes at a density of 4 × 10^4^ cells/cm^2^ and allowed to expand for 48 h (∼80% confluency). At this stage (d1 of differentiation), cultures were treated with medium comprising StemPro34 supplemented with (1:100 dilution) Matrigel and (1 ng/mL) BMP4 (R&D systems) and after 24 h (d2 of differentiation), medium comprising StemPro34 with (10 ng/mL) BMP4 and (8 ng/mL) Activin A (Life Technologies). Medium exchange was performed on d4 of differentiation using RPMI supplemented with 1xB27 (Life Technologies) and small molecule inhibitors, KY02111 (10 μM) and XAV939 (10 μM) (R&D systems). From d8 onward, cells were maintained in RPMI medium supplemented with B27 only, with medium changes every 3 days. Cardiac differentiation efficiency was accessed by using immunocytochemistry with primary mouse anti-human α-actinin antibody (No. A7811, 1:800; Sigma) dilution and secondary goat anti-rabbit Alexa633 (No. A21052, 1:400; Invitrogen), counterstaining with 0.5 μg/mL DAPI (No. D9542, 1:500; Sigma). Immunofluorescence images were captured using Operetta High-Content System (Perkin Elmer) and analyzed using Harmony High-Content Analysis Software.

#### Gene expression

RNA was isolated from undifferentiated hPSCs and derived CMs at day 14 of differentiation using RNeasy Mini Kit (Qiagen). Synthesis of cDNA was carried out using 1 μg RNA with SuperScript III Reverse Transcriptase Kit (Invitrogen), according to manufacturer's instructions. *ADRB2* analysis was with TaqMan qPCR (No. Hs00240532_s1; Applied Biosystems) and signals were normalized to *GAPDH* (No. Hs99999905_m1; Applied Biosystems) as the housekeeping gene, following the manufacturer's instructions. Semi-qPCR cycle conditions were 95°C for 2 min, 64.5°C for 30 s (*GRK5*, *ACTC1*, *RYR2*, and *ACTB*), and 72°C for 60 s, with a final elongation step of 72°C for 10 min. Each reaction used 250 ng of cDNA with Phusion polymerase (NEB) for 35 cycles. Gels were imaged with a LAS-4000 (Fujifilm) image analyzer, densitometry was carried out using FIJI, and a version of ImageJ (National Institutes of Health) and signals were normalized to *ACTB* as the housekeeping gene. Primers for expression analysis are provided in Supplementary Table S1.

#### Immunocytochemistry analysis of nuclear pluripotent markers

hPSCs were cultured at 30,000 cells/cm^2^ in Matrigel-coated 96-well plates (Perkin Elmer CellCarrier) until reaching 60% confluent before fixing with 4% PFA. Fixed cells were perforated using 0.01% Triton X-100 and 0.05% Tween 20 [(diluted in phosphate-buffered saline (PBS)]. The cells were then incubated with mouse-anti human OCT4 (C-10 clone; No. sc-5279, 1:100; Santa Cruz Biotech) and subsequent secondary antibody using goat-anti mouse Alexa488 (No. A11001, 1:1000; Invitrogen) and counterstained with 0.5 μg/mL DAPI. Immunofluorescence images were captured using Operetta High-Content System (Perkin Elmer) and analyzed using Harmony High-Content Analysis Software.

#### Flow cytometry analysis of surface pluripotent markers

To analyze surface markers, hPSCs were harvested and fixed using 4% PFA followed by incubation with phycoerythrin-conjugated SSEA-1 (eBioMC-480 clone; No. 12-4752, 1:100; ThermoFisher), SSEA-4 (eBioMC-813-70 clone; No. 12-8843, 1:200; ThermoFisher), and TRA-1-81 (TRA-1-81 clone; No. 12-8883, 1:100; ThermoFisher) antibodies for 20 min at 4°C. Cells were analyzed using an FC500 Flow cytometer (Beckman Coulter), and data were analyzed with FlowJo software.

#### Karyotyping

Metaphase spreads were prepared as previously described [20] from hPSCs after final genotype was confirmed, and karyotype analysis was performed by G-banding of 30 metaphase spreads in each sample, according to guidelines from the International System for Human Cytogenetic Nomenclature.

### Functional analysis of GRK5 hPSC-CM polymorphic variants

To measure the beat rate of CMs in real time, the CardioExcyte96 system (Nanion) was used. In brief, the 96-well sensor plates of the CardioExcyte96 were coated by incubation (1.5 h) with fibronectin at 1:100 dilution in PBS (without Ca^2+^ and Mg^2+^). CMs at d25 to d28 were dissociated and seeded onto the sensor plate at 60,000 cells/well. Plates were incubated for 48 h before changing the medium and starting the recordings according to the following timeline: 0–2 h, baseline recording; 2 h, spike with 100 nM isoprenaline; 24 h, repeat spike of isoprenaline; and 48–50 h, end of recording. Beat rate of CMs was recorded throughout the experiments at intervals of 2 to 10 min. For the nonselective beta-blocker experiment, propranolol (200 nM) was added 1 h before starting isoprenaline treatment and maintained throughout.

## Results

### Locus selection and targeting strategy for *ADRB2* (β2-adrenoceptor)

Over the course of multiple experiments in our laboratory, we observed Cas9/CRISPR gene targeting efficiencies of ∼30% (158 of 421 colonies assessed) across 12 different loci and/or hPSC lines (data not shown) when using optimized transfection conditions (Supplementary Fig. S1). In the context of the two-step *PiggyBac* process, we reasoned that the gene targeting efficiency during step 1 would be rate limiting because cassette excision should occur in most cells, provided transposase delivery is at high efficiency at the start of step 2. An alternative strategy could be to merge steps 1 and 2 of the *PiggyBac* process. This would have the advantage of not only simplifying editing but also of reducing the time to produce gene modified hPSCs by 14 days; this equates to ∼14 population doublings and 25%–30% of the whole targeting process (Fig. 1B).

To test this notion, we selected the *ADRB2* locus for several reasons. *ADRB2* encodes β2-adrenoceptor, a G-protein coupled receptor that has an N-terminal domain positioned in the extracellular compartment. In this domain, two polymorphic variants at amino acid positions p.Gly16Arg (c.*G46A*) and p.Glu27Gln (c.*G79C*) alter patient response during heart failure [21]. Thus, production of isogenic hPSC lines from which CMs can be produced would be beneficial in understanding the mechanism of these differences. We also selected this locus because it is expressed in undifferentiated hPSCs, although at much lower levels than in hPSC-CMs (Fig. 1C). This may be useful since an “open” configuration is considered to be more permissible to gene targeting [22]. However, *ADRB2* also requires a footprint-free strategy because it is a single exon gene with complex 5′ and 3′ untranslated regions, which include multiple regulatory elements and domains required for proper expression of *ADRB2* and its membrane targeting [23–25]. As such, positioning a selection cassette or a short footprint in these regions may be disruptive to cell signaling and function, even in the undifferentiated state.

**FIG. 2.**
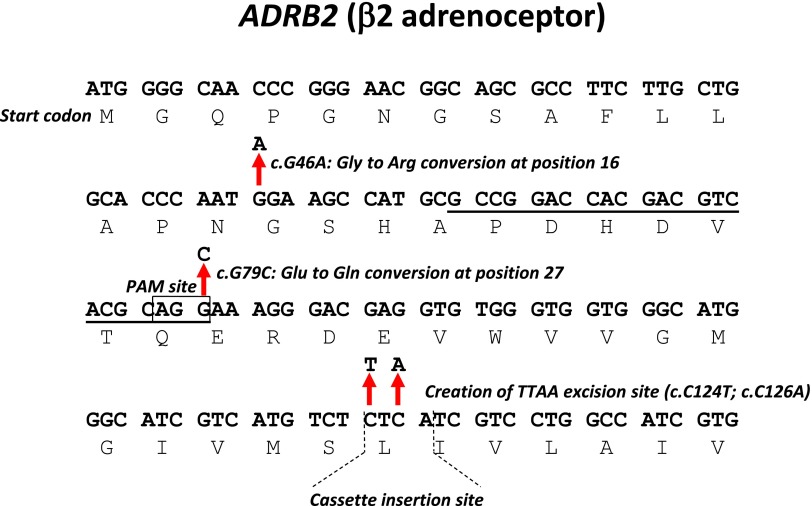
Polymorphic changes to the *ADRB2* locus in hPSCs. The nucleotide and translated single-letter amino acid sequences are shown for the 5′ region of the *ADRB2* locus. The targeting strategy introduces changes at positions 46 and 79 (nonsynonymous in the peptide), and 124 and 126 (synonymous in the peptide) as indicated. Features identified are the location of the gRNA *underlined*, with PAM site *boxed*, and *TTAA PiggyBac* cassette insertion site. PAM, protospacer adjacent motif. Color images available online at www.liebertpub.com/scd

The *PiggyBac* approach requires an endogenous quadranucleotide *TTAA* palindrome sequence at the site of recombination, which theoretically occurs at 329 bp intervals through the genome [26]. However, the *PiggyBac* transposon has a preference for areas surrounding transcription start sites and CpG islands [27], suggesting that even distribution of *TTAA* sites does not occur. Supporting this notion, our analysis of the genomic regions flanking the position 46 or 79 *ADRB2* polymorphic variants in HUES7 hESCs revealed that the nearest *TTAA* site was 748 bases away (data not shown), which far exceeds the distance recommended for insertion via nuclease-mediated targeting [10]. However, we noted the sequence *CTC ATC* (nucleotide position 124–129) situated 45 bases downstream of the position 79 polymorphism in *ADRB2* coding sequence; codon redundancy for leucine meant that substitutions could be made to *TTA ATC*, which created the *TTAA* site necessary for *PiggyBac* recombination while being synonymous and retaining the native Leu41-Ile42 peptide sequence (Fig. 2).

We sought to minimize any further changes, silent or otherwise, to the *ADRB2* locus. Therefore, we selected a gRNA with a protospacer adjacent motif (PAM) overlapping the polymorphic change c.*G79C*, ensuring cleavage of genomic, but not targeting vector, sequences would occur (Fig. 2 and Supplementary Table S1). Thus, the left arm of homology in the targeting vector contained c.*G46A* (p.Gly16Arg), c.*G79C* (p.Glu27Gln), and c.*C124T*/c.*C126A* (synonymous: p.Leu41-Ile42) modifications directed toward the *ADRB2* locus (Figs. 1A and 2).

### Simplified *PiggyBac* gene editing in *ADRB2* in hPSCs

The process outlined in Fig. 1 entails two steps, with gene targeted insertion of the *PiggyBac* cassette and the associated polymorphic changes occurring in the first step, followed by transposase-mediated cassette removal in the second step. As we anticipated that cassette excision should occur at high efficiency (Supplementary Fig. S1), we wished to test whether the frequency and types of targeting events were similar after first (midpoint) and second (end) steps. In addition, we wanted to ensure that streamlining the process by progressing directly from positive (puromycin) to negative (ganciclovir) selection did not have a detrimental effect.

**FIG. 3.**
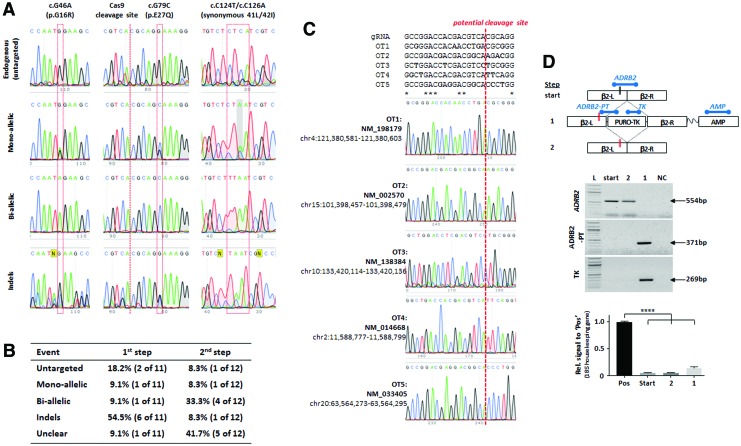
Gene editing at the *ADRB2* locus in hPSCs. **(A)** Shows representative chromatogram synopses flanking positions 46, 79, and 124–126 of untargeted, mono- and biallelic targeting, and indels. A complete set for step 1 and step 2 targeting is in Supplementary Figure S2A and B. The table in **(B)** summarizes the different targeting events identified after step 1 (midpoint; after puromycin selection for clones containing the positive–negative selection cassette) and step 2 (after ganciclovir selection for clones in which the cassette has been excised). In **(C)**, high-risk OT sites were classified as known coding or regulatory sequences where gRNAs had full PAM site complementarily and/or fewer than five mismatches with the target. PCR genotyping showed no evidence for off target events. In **(D)**, random integration was tested. The schematic shows the stages of targeting and location of PCRed regions. *ADRB2* is a control for genomic DNA, while *ADBR2-PT* and *TK* test for the presence of the targeting cassette; results are shown in the gel images. Since no product was identified for *AMP* within the pBluescript backbone, qPCR was used and compared against a positive control (pos) comprising plasmid DNA diluted to the equivalent of single-copy gene level in HUES7 parental DNA. Housekeeping gene was *18S*, *n* = 3 ± SD; ****P < 0.001, Dunnett's test. OT, off target; qPCR, quantitative polymerase chain reaction. Color images available online at www.liebertpub.com/scd

HUES7 hESCs were cotransfected with Cas9, gRNA, and *ADRB2* targeting plasmids and then subjected to puromycin treatment. Once early-stage drug-resistant colonies had formed, a portion of the colonies were picked for genotyping after step 1. The remainder of the cells were harvested, transfected with transposase, and then treated with ganciclovir, before allowing colonies to form for picking and genotyping after step 2. All clones were assessed by PCR amplification coupled to direct sequencing across the left arm of homology (Fig. 3A, Supplementary Table S1, and Supplementary Fig. S2A, B). Genotyping after first versus second step showed high frequencies (Fig. 3B), wherein genomic cleavage was evident in 8/11 (73%) and 6/12 (50%). Specifically between categories 18% versus 8% untargeted, 9% versus 8% monoallelic targeting, 9% versus 33% biallelic targeting, 55% versus 8% indels, indicated by messy reads around Cas9 cleavage site, and 9% versus 42% unclear result, indicated by PCR failure or lack of sequencing data (Fig. 3A and Supplementary Fig. S2A, B).

We also evaluated off target events (Fig. 3C and Supplementary Table S1). We focused on known coding or regulatory sequences where gRNAs had full PAM site complementarily and/or fewer than five mismatches with the target. The five putative sites that met these criteria were shown by PCR amplification and sequencing to be unaffected by off targeting (Fig. 3C). Therefore, the simplified *PiggyBac* approach was successfully used to produce an isogenic set of wild type (untargeted), heterozygote (monoallelic), and homozygote (biallelic) dual-site modifications at nucleotide positions 46 and 79 in the 5′ end of the *ADRB2* gene in hESCs.

Finally, we tested for unwanted random integration events of the vector elsewhere in the genome by PCR (Fig. 3D). As expected, control primers that spanned the PAM site in *ADRB2* gave a product from parental cells and after step 2, but not step 1, as the biallelic presence of a complex puro-ΔTK cassette blocks the PCR. Correspondingly, PCR products specific to the *ADRB2-puro-ΔTK* junction and to *ΔTK* were produced only from step 1 samples, indicating that no residual targeting selection cassette could be detected after transposase-mediated removal. Remnants of the pBluescript plasmids backbone were tested for by PCR to the ampicillin gene. No products were seen by conventional PCR (data not shown). Therefore, qPCR was carried out using a positive control, wherein targeting plasmid DNA was diluted to the equivalent of a single genomic copy into parental HUES7 DNA. Relative to this positive control, samples from parental cells, step 1 and step 2 gave a signal 10- to 20-fold lower. Collectively, these data suggest that precise targeting of the selection cassette occurred only at the *ADRB2* locus and not at random elsewhere in the genome, and cassette excision occurs after transposase-mediated removal.

### Applying simplified *PiggyBac* gene editing to other cardiac-associated loci in hPSCs

**FIG. 4.**
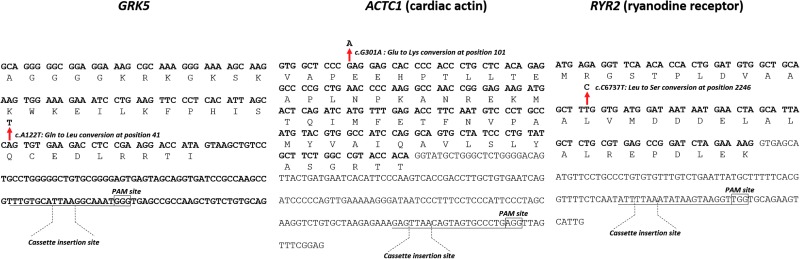
Polymorphic changes to the *GRK5*, *ACTC1*, and *RYR2* loci in hPSCs. The nucleotide and translated single-letter amino acid sequences are shown for each locus. The targeting strategy introduces nonsynonymous changes as indicated. In each case, the gRNA, with PAM site *boxed*, spans an endogenous *TTAA* cassette insertion site, which eliminates the need for changes to spare the targeting vector from Cas9-mediated cleavage. Color images available online at www.liebertpub.com/scd

Efficiency of gene targeting, including using Cas9/CRISPR, is known to be influenced by genomic environment, including complexity and GC-richness of gene sequence, active gene expression, availability of sites to guide nuclease docking, and cell type. Therefore, we selected three additional cardiac-associated loci with different genetic properties, but each with relevance to human health or heart disease (Fig. 4).

*GRK5* encodes G-protein coupled receptor-specific kinase involved in β-adrenergic receptor desensitization. It has been suggested that a c.*A122T* (p.Gln41Leu) polymorphism causes a natural β-blocker effect that may be protective against heart disease [28]. *ACTC1* encodes cardiac actin and a mutation at c.*G301A* (p.Glu101Lys) causes hypertrophic cardiomyopathy, altered calcium sensitivity, arrhythmias, and, in some cases, sudden cardiac death [29]. Finally, *RYR2* encodes ryanodine receptor, which is a calcium release channel in the sarcoplasmic reticulum. A highly malignant mutation of c.*C6737T* (p.Ser2246Leu) causes catecholaminergic polymorphic ventricular tachycardia (CPVT), which can lead to sudden cardiac death [30].

**FIG. 5.**
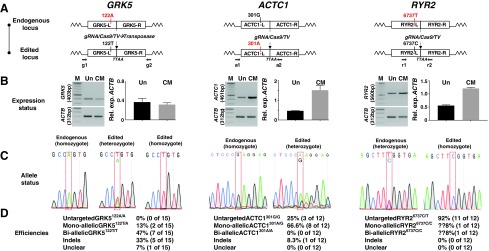
Gene editing at the *GRK5*, *ACTC1*, and *RYR2* loci in hPSCs. The schematics in **(A)** show the loci for each gene before and after editing, with damaging (*black* to *red*; *ACTC1*), protective (*red* to *black*; *GRK5*), or rescue (*red* to *black*; *RYR2*) polymorphisms introduced. L and R represent the left and right regions of homology, while primer locations for g, a, and r are indicated (full details in Supplementary Table S1). In **(B)**, semiquantitative RT-PCRs were carried out for each gene in undifferentiated hPSCs (Un) and CMs at day 30 of differentiation (CM). Bands were quantified by densitometry and normalized to β-actin (*ACTB*) as a housekeeping gene. M, marker; *n* = 2, errors are ± SD. **(C)** shows representative chromatogram synopses flanking polymorphic positions for each gene, while editing efficiencies are displayed in the tables in **(D)**. Note that for *RYR2*, it is not possible to tell whether the event was mono- or biallelic, hence the ?? symbols. RT-PCR, reverse transcription-polymerase chain reaction. Color images available online at www.liebertpub.com/scd

All four genes were expressed in undifferentiated hPSCs (Figs. 1 and 5), which is surprising since *ACTC1* encodes for cardiac actin, a CM-specific structural protein (Fig. 5). The GC content of the region surrounding the polymorphisms, gRNA and *TTAA* sites differs between *ADRB2* (64%), *GRK5* (56%), *ACTC1* (53%), and *RYR2* (42%) (Figs. 2 and 5). Thus, this set provided an opportunity to test the simplified *PiggyBac* approach in genes differing in sequence composition and that were expressed at relatively low levels in hPSCs.

Each gene was targeted in a different hPSC line out of necessity. The starting genotypes were hESC (line HUES7) *GRK5^122A/A^*, hiPSC *ACTC1^301G/G^* from a healthy individual within a family with familial hypertrophic cardiomyopathy, and hiPSC *RYR2^6737C/T^* from a young patient with CPVT. In designing the targeting strategies (Fig. 5), we elected to use endogenous *TTAA* sites for *PiggyBac* recombination that resided in neighboring introns. In addition, for each of the three genes (*GRK5*, *ACTC1*, and *RYR2*), gRNAs were chosen that spanned these *TTAA* sites; this means that the gRNAs recognized the endogenous genomic sequence but not the targeting vector because the *TTAA* demarcates the *PiggyBac* cassette insertion site (Fig. 5).

Adopting these two strategies allowed production of true isogenic lines; that is, no further sequence changes with potentially unknown effects were required either to form a de novo *TTAA* site or to protect the targeting vector from gRNA/Cas9 cleavage. The potential disadvantage of this approach is that the distance between gRNA/Cas9 cleavage site and the desired polymorphic change is increased, which raises the likelihood of recombination occurring between these two locations and hence not carrying the polymorphic change into the genome. Indeed, while the distance between PAM site and polymorphic change was 107 and 136 bp for *GRK5^122A/A^* and *RYR2^6737C/T^*, respectively, it was 313 bp for *ACTC1^301G/G^* (Fig. 4).

Targeting vectors were constructed for these genes (Figs. 4 and 5A) using the same design principles that were used for *ADRB2* and thus relied on ∼2 kb of total homology, with ∼1 kb in each of the left and right arms (Fig. 1). Following positive (puromycin) and then negative (ganciclovir) selection, colonies were expanded for PCR and sequence analysis (Fig. 5C). For all three genes, successful targeting of the polymorphisms to the left arms was observed with concurrent excision of the *PiggyBac* selection cassette and reconstitution of the endogenous *TTAA* site (Fig. 5C, D). However, the targeting efficiencies differed considerably (Fig. 5D). In *GRK5*, genomic cleavage was confirmed in 93% clones, of which 13% and 47% were monoallelic and biallelic targeting events, respectively. This overall trend of correct targeting was similar to *ACTC1*, where cleavage was 75%, although this led to 67% and 0% monoallelic and biallelic targeting events, respectively. In contrast, cleavage was only evident in 8% of *RYR2* clones, which converted to a successful editing event. In summary, the simplified approach was used to produce footprint-free, isogenic pairs for four cardiac-related genes in hPSCs.

### Characterization of gene edited hPSCs

**FIG. 6.**
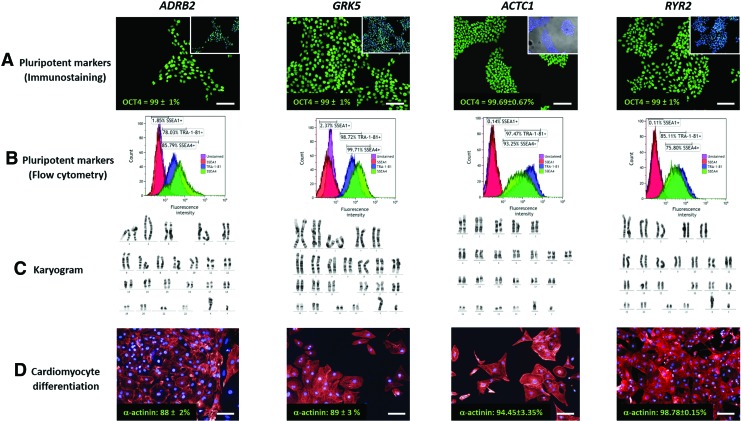
Retention of pluripotency characteristics in the edited hPSC lines. **(A–C)** Show assessment of pluripotency characteristics in undifferentiated cells from each of the edited lines. This included **(A)** immunostaining for the transcription factor, OCT4 [*green*; *inset* with DAPI (*blue*) counterstaining], **(B)** flow cytometry for TRA-1-81 (*blue*), SSEA4 (*green*), and SSEA1 (*red*), relative to unstained (*purple*), and **(C)** G-banding karyotyping of 30 metaphase spreads per line, with a representative karyogram shown for each. In **(D)**, directed monolayer differentiation produced CMs of >88% purity, as gauged by immunostaining for α-actinin (*red*) relative to total nuclei count (DAPI, *blue*). Scale bar is 100 μm; *n* = 2–4, SD. Color images available online at www.liebertpub.com/scd

Although correct targeting had been achieved, it was important to confirm whether specific pluripotency and differentiation characteristics were retained in *ADRB2*, *GRK5*, *ACTC1*, and *RYR2* gene-edited hPSC lines. Representative examples are shown (Fig. 6), but similar results were obtained from multiple clones, with the exception of *RYR2* where only one successful targeting event was identified. In all cases, immunostaining coupled with high-content image analysis showed that almost all cells expressed the pluripotency marker, OCT4. This was supported by flow cytometry, where 78%–99% and 76%–100% of hPSCs being positive for TRA-1-81 and SSEA4, whereas <3% displayed the differentiation marker, SSEA1 (Fig. 6).

The metaphase spreads of 30 cells per line were assessed by G-banding karyotyping. Assembly of homologous chromosomes into a karyogram showed no evidence of aberration. Finally, directed monolayer differentiation was used on each line to induce beating sheets of CMs. These were dispersed on day 12–15 of differentiation and stained with α-actinin, before using high-content image analysis to show CM purity was between 88% and 98%. Thus, the edited lines retained key characteristics of pluripotency, most notably differentiation to functional CMs.

### Evaluating consequences of GRK5-L41 and –Q41 variants on hPSC-CM function

**FIG. 7.**
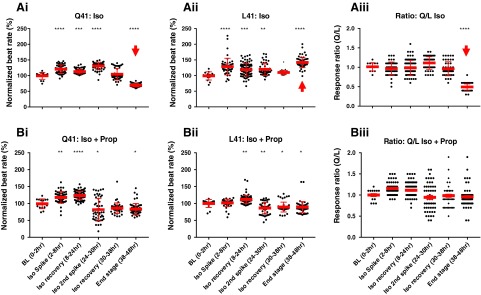
Functional effects of chronic isoprenaline on GRK5-L41 and –Q41 hPSC-CMs. Using the CardioExcyte impedance platform, the beat rate of the edited hPSC-CM lines was monitored at ∼10 min intervals during chronic stimulation (∼50 h) with 100 nM isoprenaline (Iso; **Ai**, **Aii**) with or without beta-blockade with 200 nM propranolol (Prop; **Bi**, **Bii**). Data were binned for the periods shown and plotted as normalized to percent change from BL. The response ratios were calculated by dividing each datum from GRK5-Q41 by the corresponding time point from GRK5-L41 hPSC-CMs without (**Aiii**) or with (**Biii**) blockade with propranolol. *Arrowhead* indicates where there is a highly significant decline in the beat rate of the GRK5-Q41 hPSC-CMs. Dunnett's test relative to BL: *P < 0.05; **P < 0.01; ***P < 0.001; ****P < 0.0001. BL, baseline. Color images available online at www.liebertpub.com/scd

To demonstrate the utility of isogenic sets of hPSC lines, we selected wild-type GRK5^122A/A^ and homozygote-edited GRK5^122T/T^ lines, which differ only in leucine (L) or glutamine (Q) at position 41 of the encoded peptide. It has been suggested that the GRK5-L41 variant acts as a natural β-blocker and so is protective against adrenergic stress in the heart [28]. Therefore, we seeded confluent monolayers of CMs derived from the GRK5 isogenic lines onto the CardioExcyte-96 impedance platform to assess beating characteristics during chronic (up to 50 h) stimulation with the β-adrenoceptor agonist, isoprenaline (Fig. 7).

During the first 30 h of isoprenaline treatment, CMs from both variants showed similar responses with maximum beat rates reaching ∼150% of baseline values (Fig. 7Ai, Aii). This similarity was confirmed by calculating normalized beat rate (GRK5-Q41 divided by GRK5-L41), which gave values of close to 1 (Fig. 7Aiii). However, from 30 h onward, the normalized rate of GRK5-Q41 declined, finally reaching 60%–80% of baseline by the 38–48 time window. In contrast, by the end of the evaluation period, GRK5-L41 maintained an average rate of 150%, which was reflected in a Q41/L41 response ratio of ∼0.5 (note arrows in Fig. 7Ai–Aiii). This mirrors in vivo findings, which show that, unless compensation mechanisms can be invoked, prolonged (>30 h) activation of adrenoceptors by catecholamines compromises CM recovery [28].

Since the GRK5-L41 variant has been suggested to impart a mild protective effect during chronic β-adrenergic stimulation, we reran the experiment but this time with coincubation of isoprenaline and the nonspecific β-blocker, propranolol (Fig. 7B). As expected, the initial chronotropic response of both variants was subdued by propranolol. Notably, however, the chronic decline in beat rate to well below baseline levels seen by 38–48 h in GRK5-Q41 with isoprenaline alone (Fig. 7Bi) was abolished with the addition of propranolol (Fig. 7Bii) and was reflected by response rate ratios of close to 1 throughout the timecourse (Fig. 7Biii). Thus, chronic overstimulation of the β-adrenoceptor system eventually caused a decline in beat rate in GRK5-Q41, but not GRK5-L41 hPSC-CMs, and this could be reversed by β-blockade. This provides a tool for mechanistic understanding of genotype–phenotype interactions, which we are now investigating.

## Discussion

We successfully demonstrated a simplified footprint-free approach to gene edit four distinct cardiac-associated loci in hPSCs, with modifications, including mono- and/or biallelic targeting. This included introducing polymorphic changes in hESC and/or hiPSC lines that were anticipated to be mildly beneficial to CM function into *ADRB2* and *GRK5* or severely damaging into *ACTC1*. We also corrected a damaging mutation in the *RYR2* gene. The edited hPSC lines retained the ability to undergo high efficiency differentiation to CMs, enabling us to demonstrate the utility of this approach by showing functional differences in drug response for the GRK5 isogenic set. This simplified *PiggyBac* approach is easily adaptable to other loci, providing there is appropriate proximity of *TTAA* sites, either native or modified by engineering. Applicability will be irrespective of whether the targeting strategy uses conventional or nuclease (eg, zinc fingers, TALE, Cas9/CRISPR) strategies and will be of future value in facilitating mechanistic studies.

The need for isogenic hPSC lines was highlighted recently by Sala et al. [3]. Comparison of action potential duration 90 (APD_90_), an electrophysiology parameter, in CMs derived from 18 hPSC lines showed more than a fourfold difference, with values ranging from ∼140 to 600 ms. Even between different commercial suppliers of hPSC-CMs, where quality control is high before release to customers, the range was 225 to 600 ms. A notable departure from this variation was one isogenic pair, where CMs from both lines had highly similar APD_90_ values of ∼230 ms.

Contextually, the normal range for humans APD_90_ values (usually cited as QT interval) is 350–450 ms, and increases of 10%–20% are worrisome. During drug development, such prolongation would likely lead to the termination of the drug [31]. Clinically, QT intervals of >460–500 ms usually signify disease state, such as long QT syndrome, which is caused by mutations in various ion channel proteins and can lead to sudden cardiac death [32]. This means that depending on the hPSCs selected, the phenotypic variation between lines (up to 400%) can be greater than any change caused by the mutation (usually 10% to 100%). This may explain some of the discrepancies reported in the literature for hPSC-based disease modeling, including for the magnitude of change caused by mutations in *KNCQ1*, which underlies long-QT syndrome type 1 [33,34]. Consequently, the use of isogenic pairs is becoming the gold standard for disease modeling using hPSCs. The isogenic approach allows desired polymorphisms to be studied within the same genetic background and the “noise” is eliminated from the other estimated ∼11 million single nucleotide polymorphisms (SNPs), 2.8 million short indels, and ∼500,000 block substitutions that exist between unrelated individuals [35].

A true isogenic pair will differ only in the desired polymorphic change. Part or whole remnants of selection cassettes can perturb gene function [36], even when positioned in introns because of the presence of currently unannotated sequences. Indeed, in hPSCs, we found that even when Cas9/CRISPR was used to target Ef1α-driven blasticidin or puromycin resistance markers into neighboring introns, this abolished expression of *KCNH2* [1] and *MYH7* (Supplementary Fig. S3C) genes encodes the HERG potassium ion channel and beta myosin heavy-chain structural protein, respectively. In both cases, cassette removal restored expression of *KCNH2* and *MYH7*. For *ADRB2*, the complexity of the locus and absence of a nearby *TTAA* site necessitated conversion of *CTC ATC* to *TTA ATC*. In humans, both *CTC* and *TTA* are compatible with the leucine tRNA machinery but the probability of use is 0.2 and 0.07, meaning that *CTC* is preferred. Also, current gene annotation shows this change should not interfere with control regions (promoters, enhancers, noncoding RNAs, splice sites, etc.), but needs to be borne in mind during targeting design. Thus, any changes, from single bases through to residual sequences ([1,37,38]; Supplementary Fig. S3), may need thorough investigation to rule out any potential negative impact on cell function.

For loci that are more refractory to targeting, cultures can be pooled at the midpoint of the process (after step 1/puromycin treatment) and an aliquot of cells taken for bulk PCR analysis. Primers are chosen to span from the selection cassette to the flanking genomic region of the locus of interest. If no PCR product is produced, this may suggest that the experiment should be abandoned. However, if there is a product, then, the cells can be reseeded, transfected with transposase, and then treated with ganciclovir to finish the excision/colony selection process.

A surprising observation was that when cells at this puromycin-resistant midpoint were cryopreserved, the positive–negative selection cassette was silenced upon thawing of the cells; this occurred across several loci beyond those described in this report. We are unsure as to why the cryopreservation–thaw cycle caused this effect. Indeed, it is well documented that silencing of transgenes occurs readily in hPSCs, particularly when nonmammalian promoters are used [39]. However, we used the mammalian promoter, phosphoglycerate kinase, which is usually well tolerated [40,41]. We are not aware of other reports where transgene expression is maintained during long-term culture, unless a cryopreservation–thaw cycle is introduced.

Although all loci were targeted successfully, there were notable differences. Genome editing occurred at an efficiency of 42%–67% in *ADRB2*, *GRK5*, and *ACTC1*, but only 8% in *RYR2*. All the genes were expressed, but this is not a prerequisite for Cas9/CRISPR targeting. Our data for *MYH7* showed a frequency of mono- and biallelic events totaled ∼25% (Supplementary Fig. S3). In terms of GC content, *RYR2* had the lowest (42%) around the target site, which might be expected to give better access for gene targeting rather than the lowly 8% reported in this study. This may be because the complexity of the *RYR2* locus is high, with regions flanking the target site, including repetitive elements (LINE, SINE, Alu). Another parameter that could influence targeting efficiency is the cell line used. Out of necessity, we used different hPSC lines because of their starting genotype, which in some cases was disease- or patient-specific. Many similarities and differences have been reported between hPSC lines [16]. In our report, we found that the puromycin concentration required during selection varied from 0.25 to 7.5 μg/mL. Thus, it would be unsurprising if variation extended to differential targeting efficiencies between hPSC lines.

Vector construction and lengths of homology regions are also factors known to impact targeted recombination [42]. The same design principles were used for all four loci, but the distances between the PAM site in the gRNA and polymorphism (termed PAM-SNP) varied out of necessity. Differences in targeting frequency may be explained by the mechanism of repair. DNA repair occurs via multiple pathways or subpathways, including DNA double-strand break repair (DSBR), Holliday junction dissolution, synthesis-dependent strand annealing, and single-strand DNA incorporation [43,44]. With regard to DSBR, long conversion tracts (approximately ±1 kb) are generated either side of the of conversion zone, with probability of conversion decreasing as a function of PAM-SNP distance [45]. Linear dependency also occurs with ssODNs [9,43], but creates conversion tracts of approximately ±60 nucleotides [43], which is why this approach tends to only incorporate small insertions or substitutions.

The PAM-SNP frequency-distance relationship may explain some of the differences in nature and efficiency of targeting events. For *ACTC1*, with a 300 nucleotide distance, there was a higher probability of recombination occurring between PAM site and polymorphism. After transposase-mediated cassette excision, the sequence in the final chromatogram would appear as untargeted because the approach was designed to be footprint-free. This may have contributed to a profile of clones being untargeted = high (25%), monoallelic targeted = high (67%), and biallelic targeted = low (0%). In contrast, the short PAM-SNP distance of around 100 nucleotides or less for *ADRB2* and *GRK5* presented profiles of 0%, 8%, and 33% and 8%, 13%, and 47%, respectively. Fortuitously, only heterozygote mutations occur in humans for *ACTC1*, presumably because it would likely lead to early lethality, which is the case in mouse knockouts. However, the PAM-SNP distance is clearly not the only factor, since most (92%) clones for *RYR2* were not targeted. We cannot be sure whether the one *RYR2* clone was mono- or biallelic targeting event since the template was identical to the healthy allele, so only correction of the mutant allele could be detected.

Our main goal in this work was to reduce the duration required to produce isogenic sets of hPSCs, with a specific emphasis on in vitro disease modeling of the cardiovascular system. While others have used the *PiggyBac* system, we describe an abbreviated version that not only saves time and effort but also the number of population doublings required to produce the gene-edited cells. This is important because both empirical experimentation [14] and mathematical modeling [46] show that genetic and epigenetic changes are inevitable as a function of time.

The targeted clones in this study were examined by karyotyping of at least 30 metaphase spreads. Nevertheless, further detailed analysis will be needed to examine the broader stability of these lines. The rate of epigenetic change is highest soon after hESC line derivation, with most changes being haphazard [14,15]. In contrast, many genetic changes are predictable. This is exemplified by a large-scale study [14] of 136 hESC and hiPSC lines from 38 laboratories worldwide, which showed a progressive tendency to acquire changes on prolonged culture. Common changes at the chromosome level were part or whole gains of 1, 12, and/or 17. However, in ∼20% of lines studied, there was also gain of a minimal amplicon in chromosome 20q11.21. This included three genes, *ID1*, *BCL2L1*, and *HM13*, with *BCL2L1* driving a selective advantage for hPSC survival in culture. Whether stochastic or nonstochastic, these changes may affect the quality of the cells for biomedical application. Strategies to reduce the population doublings required during their manipulation should be welcomed, although to date this has not been considered. This would bring genetically engineered hPSCs into kilter with the international guidelines for clinical grade lines, where low-passage seed stocks or master banks are recommended [47].

## Supplementary Material

Supplemental data

Supplemental data

Supplemental data

Supplemental data
