# Supplementary material for: Simplified Footprint-Free Cas9/CRISPR Editing of Cardiac-Associated Genes in Human Pluripotent Stem Cells
Source: Stem Cells Dev. 2018 Mar 15;27(6):391–404. doi: 10.1089/scd.2017.0268 (PMC5882176; doi:10.1089/scd.2017.0268)
Supplement: Supplemental data [file Supp_Table1.pdf]

Supplementary Table S1. Primers for Vector Construction and Genotyping

Target	*Accession*	*Reagent*	*Use*	*Forward/ reverse*	*5′ → 3′ sequence*	*Product size (bp)*	*See Fig.*
ADRB2	NM_000024	LH primer	VC	F	GGTCGACGGTATCGATAAGCTTGATT TCGGAGTACCCAGATGGAGAC	1084	Fig. 1A
				R	ACGCAGACTATCTTTCTAGGGTTAA AGACATGACGATGCCCATGC		
		RH primer		F	CAATATGATTATCTTTCTAGGGTTAA TCGTCCTGGCCATCGTGTTTGG	1083	
				R	ATCCCCCGGGCTGCAGGAATTCGATA GTCTTCCGTGCCTGGGAGGTC		
		ADRB2puroΔtk	CA	F	CATCGTCATGTCTTTAACCCTAGAAA GATAGTCTGCG	3267	
		puroΔtkADRB2		R	ACACGATGGCCAGGACGATTAACCCT AGAAAGATAATCATATTGTGACG		
		Primer b1	Genotyping	F	GCTCGGGTGAGGCAAGTTCGG	1331	
		Primer b2		R	ATGGCAAAGTAGCGATCCAC		
		Primer b3	Expression	F	GCTGAGTGTGCAGGACGAGT	555	Fig. 5
		Primer b4		R	ATGGCAAAGTAGCGATCCAC		
		gRNA	Targeting	+ Strand	GCCGGACCACGACGTCACGC	n.a.	Fig. 2
	NM_198179	Primer OT1	OT1	F	CCTGAGCTGCTCTCCTTTCC	378	Fig. 3C
				R	CCAGAGCATTGCCAAAGAGC		
	NM_002570	Primer OT2	OT2	F	CAGCATGGAGAAGAGGAGCC	387	
				R	ACTGTCACCCTTGTCCCAGA		
	NM_138384	Primer OT3	OT3	F	GATGCCATCATGGAGCCTCT	357	
				R	ACCCTAGTGACCAGCATGGA		
	NM_014668	Primer OT4	OT4	F	TGCACTCAATGAGCAAGGCT	344	
				R	CCCAGCTGGACCAGGTAGTA		
	NM_033405	Primer OT5	OT5	F	GAGGAGGTGATCAGGCAGC	332	
				R	TCCCCATGCTTCTCACACAG		
	NM_000024	ADRB2	Random integration	F	GCTGAGTGTGCAGGACGAGT	554	Fig. 3D
				R	ATGGCAAAGTAGCGATCCAC		
	n/a	ADRB2-PT		F	GCTGAGTGTGCAGGACGAGT	371	
				R	CTAAATGCACAGCGACGGATTCGCGC		
		TK		F	ATAGACGGTCCTCACGGGAT	269	
				R	ATATGAGGAGCCAGAACGGC		
		AMP		F	CTGCAATGATACCGCGAGAC	576	
				R	TCCTTGAGAGTTTTCGCCCC		
GRK5	NM_005308	LH primer	VC	F	GGTCGACGGTATCGATAAGCTTGATT AAGCGCCACTGTAAGGGTGGAGAG	1156	Fig. 5
				R	ACGCAGACTATCTTTCTAGGGTTAAT GCACAAACGGCTTGGCGGATCACC		
		RH primer		F	CAATATGATTATCTTTCTAGGGTTAA GGCAAATGGGTGAGCCGCCAAGCTG	1146	
				R	AGTGGATCCCCCGGGCTGCAGGAATT CGATGGCACAGATGGCCTCCTATC		
		Primer g1	Genotyping	F	CAGAGCAAGGTGGAGGACAG	1446	
		Primer g2		R	GATAGGAGGCCATCTGTGCC		
		Primer g3	Expression	F	TCCGAAGGACCATAGACAGAGA	403	
		Primer g4		R	TGCCTTTCCAACCACTTCCA		
	WGE ID: 1075072174	gRNA 2a	Targeting	- Strand	TTTGTGCATTAAGGCAAATG	n.a.	Fig. 4
	WGE ID: 1075072180	gRNA 2b		+ Strand	GTGAGGCAAATGCCAATCAG		
ACTC1	NM_005159	LH primer	VC	F	GGTCGACGGTATCGATAAGCTTGAT GCCAGACAGGCTGCCAAGCAGG	1050	Fig. 5
				R	ACGCAGACTATCTTTCTAGGGTTAAC TCTTTCTCTTAGCACAGAC		
		RH primer		F	CAATATGATTATCTTTCTAGGGTTAAC AGTAGTGCCCTGAGGTTAGTTT	1051	
				R	ATCCCCCGGGCTGCAGGAATTCGATG CTGGAAGAGTGTCTCAGGACAG		
		Primer a1	Genotyping	F	CACCTGACCCTCTTGTTCGA	2267	
		Primer a2		R	GCGGATTCAGTGAGAGAGGA		
		Primer a3	Expression	F	GGTGATGAAGCCCAGAGCAA	461	
		Primer a4		R	GTGGTGACAAAGGAGTAGCC		
		gRNA	Targeting	+ Strand	GAGTTAACAGTAGTGCCCTG	n.a.	Fig. 4

(*continued*)

Supplementary Table S1. (Continued)

Target	*Accession*	*Reagent*	*Use*	*Forward/ reverse*	*5′ → 3′ sequence*	*Product size (bp)*	*See Fig.*
RYR2	NG_008799.2	LH primer	VC	F	ACGGTATCGATAAGCTTGATTACGT AAAATTAAACTTTAA	360	Fig. 5
				R	GACTATCTTTCTAGGGTTAAAATATT GAGAAAACCGTGAA		
		RH primer		F	TGATTATCTTTCTAGGGTTAATATAA GTAAGGTTGGTGCA	1041	
				R	CCGGGCTGCAGGAATTCGAT CAGTAAAGGAAACAGGAAGA		
		Primer r1	Genotyping	F	CCCCAGCTATGAGAGGTTCA	445	
		Primer r2		R	GAACGTTGGTTCTCCTTCCA		
		Primer r3	Expression	F	TGCATGAAAGCATCAAACGCA	560	
		Primer r4		R	TGAGTAGAGCCGGAGAGTGT		
		gRNA	Targeting	+ Strand	ATTTTAAATATAAGTAAGGT	n.a.	Fig. 4
ACTB	NM_001101.3	Primer GAP1	Expression	F	CAAGAGATGGCCACGGCT	312	Fig. 5
		Primer GAP2		R	CTTGATCTTCATTGTGCTGGG		

CA, cassette amplification; LH, left homology; RH, right homology; VC, vector construction.
