# Supplementary material for: Simplified Footprint-Free Cas9/CRISPR Editing of Cardiac-Associated Genes in Human Pluripotent Stem Cells
Source: Stem Cells Dev. 2018 Mar 15;27(6):391–404. doi: 10.1089/scd.2017.0268 (PMC5882176; doi:10.1089/scd.2017.0268)
Supplement: Supplemental data [file Supp_Figure1.pdf]

Supplementary Data

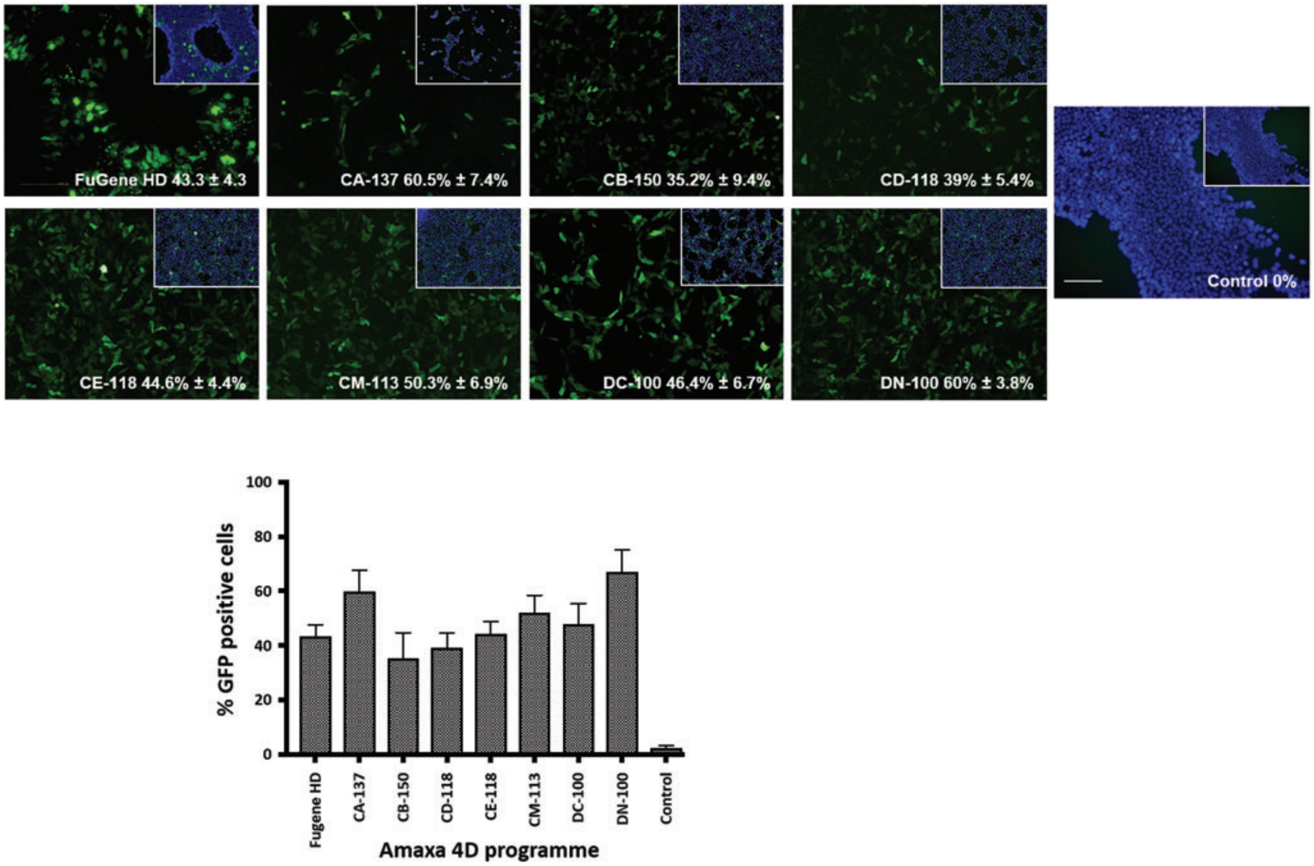

SUPPLEMENTARY FIG. S1. Optimization of transfection conditions for gene editing in hPSCs. Transfection efficiency of a GFP-expressing plasmid into undifferentiated HUES7 hESC was evaluated using chemical transfection with Fugene HD or using various programs embedded in the Amaxa 4D nucleofection system. Shown are representative fields of GFP (*green*; *inset* is with DAPI [*blue*]), which were quantified by high-content image analysis using the Operetta confocal plate reader. Data are $n = 3$, mean ± SEM. Scale bar is 100 μm. GFP, green fluorescent protein; hESCs, human embryonic stem cells; hPSCs, human pluripotent stem cells.
