# Supplementary material for: Simplified Footprint-Free Cas9/CRISPR Editing of Cardiac-Associated Genes in Human Pluripotent Stem Cells
Source: Stem Cells Dev. 2018 Mar 15;27(6):391–404. doi: 10.1089/scd.2017.0268 (PMC5882176; doi:10.1089/scd.2017.0268)
Supplement: Supplemental data [file Supp_Figure2.pdf]

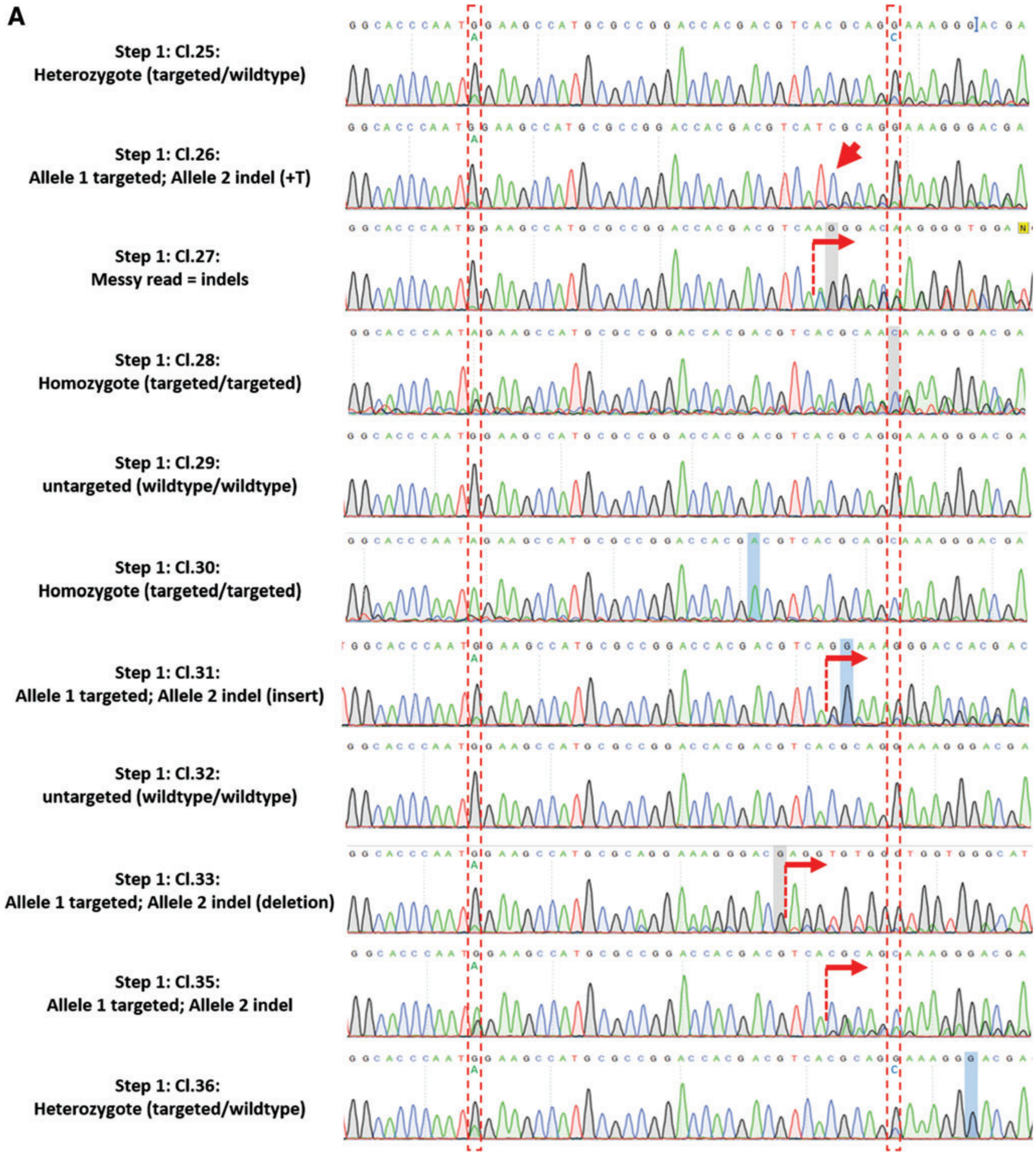

SUPPLEMENTARY FIG. S2. **(A, B)** Sequencing data for *ADRB2* editing. Chromatograms derived from direct sequencing of PCR products are shown for the clones picked after step 1 (Fig. 2A; midpoint; after puromycin selection) and after step 2 (Fig. 2B; end; after ganciclovir selection and cassette excision). In **(A)** (read in forward direction), the polymorphic sites at nucleotide positions 46 and 79 are shown, but not the *TTAA* site since this is disrupted by cassette insertion. In **(B)** (read in reverse direction), the polymorphic sites at nucleotide positions 46 and 79, as well as the reconstituted *TTAA* site. Heterozygote (monoallelic) and homozygote (biallelic) targeting events are shown, with the former indicated by double peaks and both bases shown. *Arrows* indicate region where sequence becomes misaligned due to indels. PCR, polymerase chain reaction.

(*continued*)

B

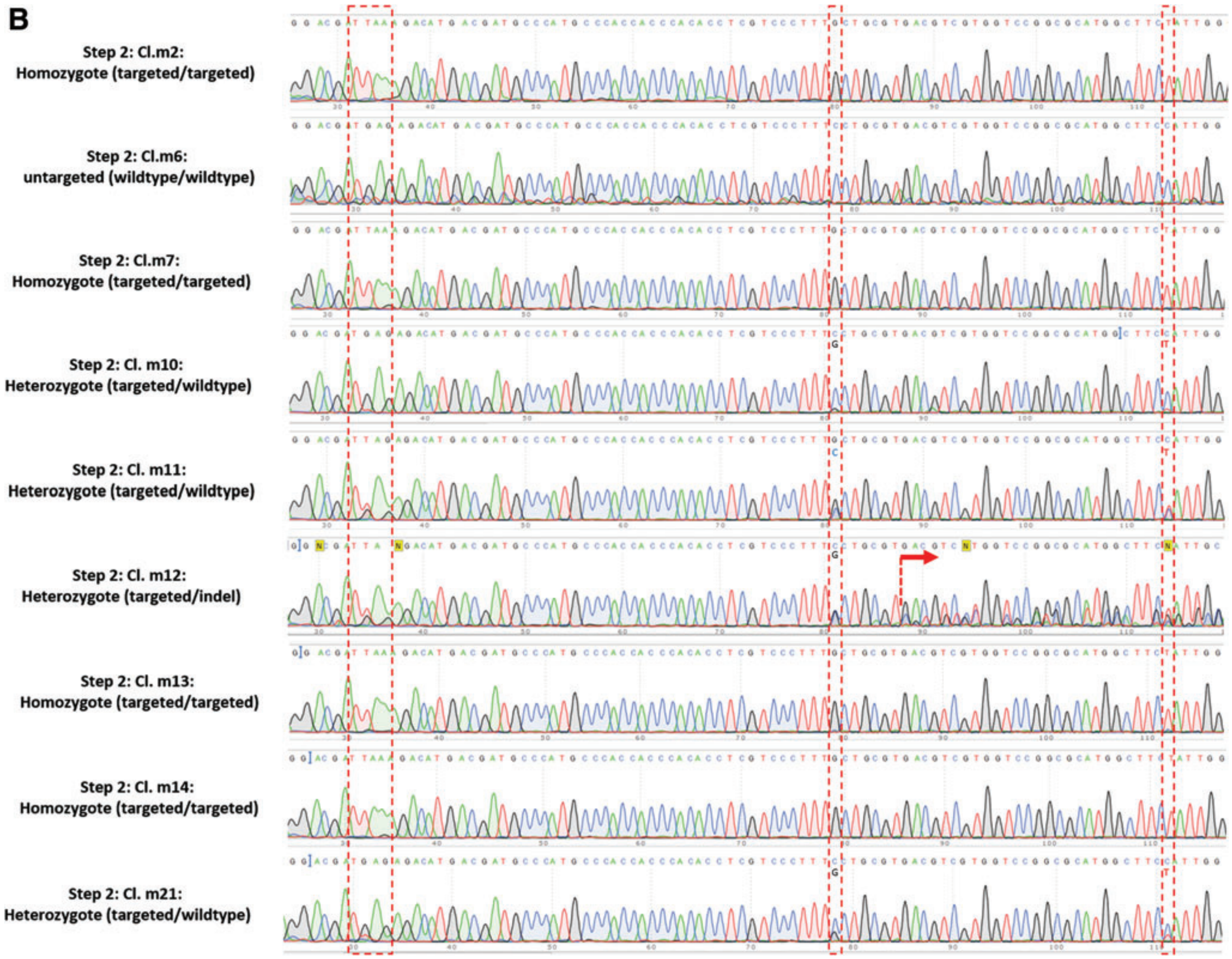

SUPPLEMENTARY FIG. S2. (Continued).
