# Supplementary material for: Simplified Footprint-Free Cas9/CRISPR Editing of Cardiac-Associated Genes in Human Pluripotent Stem Cells
Source: Stem Cells Dev. 2018 Mar 15;27(6):391–404. doi: 10.1089/scd.2017.0268 (PMC5882176; doi:10.1089/scd.2017.0268)
Supplement: Supplemental data [file Supp_Figure3.pdf]

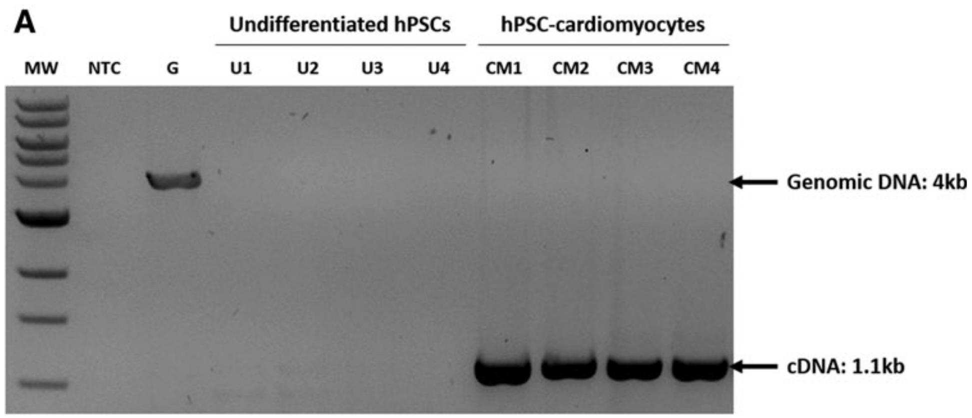

B

Untargeted	71% (169 of 236)
Mono-allelic	22% (51 of 236)
Bi-allelic	2% (4 of 236)
Indels	5% (12 of 236)

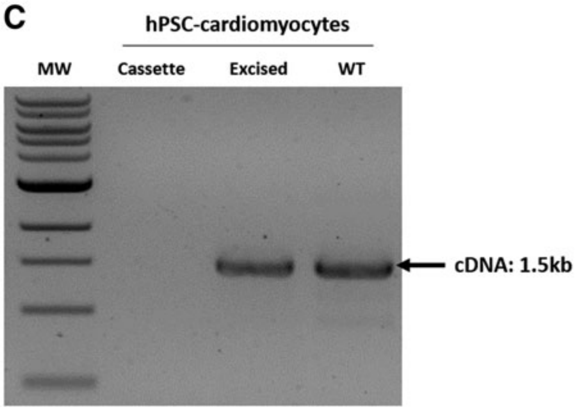

SUPPLEMENTARY FIG. S3. Gene editing in the *MYH7* locus (β-myosin heavy chain). **(A)** Shows RT-PCR analysis of *MYH7* in different samples of hPSC lines in undifferentiated state (U1-U4) or after directed monolayer differentiation to cardiomyocytes (CM1–CM4); only the latter shows expression. Despite the lack of gene expression in the undifferentiated hPSCs, **(B)** shows the targeting efficiencies of ~25% after insertion of a selection cassette flanked by FRT recombination sites. In **(C)**, note that by RT-PCR *MYH7* expression occurs in WT cells or targeted cells from which the selection cassette has been removed with FLP recombinase (excised) but not when the selection cassette is present (cassette), even though it is positioned in an intron away from any annotated elements or splice junctions. FLP, flippase recombinase; FRT, FLP recombinase target sequences; G, genomic DNA; MW, molecular weight marker; NTC, no template control; RT-PCR, reverse transcription-polymerase chain reaction; WT, wild type.
